# Functional Approaches in Promoting Vascularization and Angiogenesis in Bone Critical-Sized Defects via Delivery of Cells, Growth Factors, Drugs, and Particles

**DOI:** 10.3390/jfb14020099

**Published:** 2023-02-13

**Authors:** Ghazal Shineh, Kishan Patel, Mohammadmahdi Mobaraki, Lobat Tayebi

**Affiliations:** 1 School of Biomedical Engineering, University of Sydney, Sydney, NSW 2006, Australia; 2 School of Dentistry, Marquette University, Milwaukee, WI 53207, USA; 3 Biomaterial Group, Faculty of Biomedical Engineering, Amirkabir University of Technology, Tehran 15916-34311, Iran

**Keywords:** critical-sized defect, vascularization, angiogenesis, osteogenesis, orthopedic, agent delivery

## Abstract

Critical-sized bone defects, or CSDs, are defined as bone defects that cannot be regenerated by themselves and require surgical intervention via employing specific biomaterials and a certain regenerative strategy. Although a variety of approaches can be used to treat CSDs, poor angiogenesis and vascularization remain an obstacle in these methods. The complex biological healing of bone defects depends directly on the function of blood flow to provide sufficient oxygen and nutrients and the removal of waste products from the defect site. The absence of vascularization can lead to non-union and delayed-union defect development. To overcome this challenge, angiogenic agents can be delivered to the site of injury to stimulate vessel formation. This review begins by introducing the treatment methods for CSDs. The importance of vascularization in CSDs is subsequently highlighted. Delivering angiogenesis agents, including relevant growth factors, cells, drugs, particles, cell secretion substances, their combination, and co-delivery to CSDs are fully explored. Moreover, the effects of such agents on new bone formation, followed by vessel formation in defect areas, are evaluated.

## 1. Introduction

Therapeutic options for the management and restoration of large bone defects caused by trauma, tumor ablation, or metabolic diseases are still limited. Bone defects larger than two times the diameter of the long bone diaphysis are critical-sized defects (CSD) [1]. Despite bone tissues’ capability of healing without the development of a fibrous scar [2], critical-sized bone defects cannot be recovered without surgical intervention [1]. For the in vivo assessment of different CSDs treatment strategies, models have been described for small (i.e., rat, rabbit) and large (i.e., dog, sheep, goat, and pig) animals [3]. Although the minimum size of the osseous wound that renders a defect “critical” is not well understood, a group of papers defined CSD as a segmental bone deficiency of a length exceeding 2–2.5 times the diameter of the affected bone [4,5]. The threshold size of a critical defect varies between patients and depends on the nature of the injury and its location in the body [6]. Typically, bone defects greater than 2 cm are considered CSD in the human body since, after 52 weeks, no mineralized area of ≥30% appears [6]. However, the size of CSDs for in vivo animal models depends on the type of species and the location of the defect. Table 1 represents the size of the CSD in different animal models and the location of the defect according to a group of literature.

To manage large-scale defects, different approaches can be used as treatment, including (1) application of bone grafts (autogenous, allogeneic, xenogeneic, and tissue engineering bone grafts), (2) microvascular free flap surgery, (3) induced membrane technique, and (4) distraction osteogenesis [17,18]. However, poor angiogenesis and vascularization of CSDs remain an obstacle [19]. Vascularization is defined as blood vessels growing into tissue [20]. In contrast, angiogenesis is related to new blood vessel formation through the migration, differentiation, and growth of endothelial cells [21].

One solution to resolve the challenge of poor angiogenesis and vascularization in CSDs is delivering stimulator agents to the defective site [22]. These agents can promote vessel formation in CSDs and can be considered a reconstructive technique in healing very large bone defects.

This paper reviews the CSD treatment methods. The importance of vascularization in CSDs is comprehensively explained. Moreover, vascularization and angiogenesis agents’ delivery and their effect on both vessel formation and subsequent bone formation are evaluated. The agents for improving the vascularization and angiogenesis of CSDs reviewed in this article include growth factors, drugs, cell secretory substances, and their combination as multiple treatments or co-delivery.

## 2. Treatment Methods for Critical-Sized Defects

**Figure 1:**
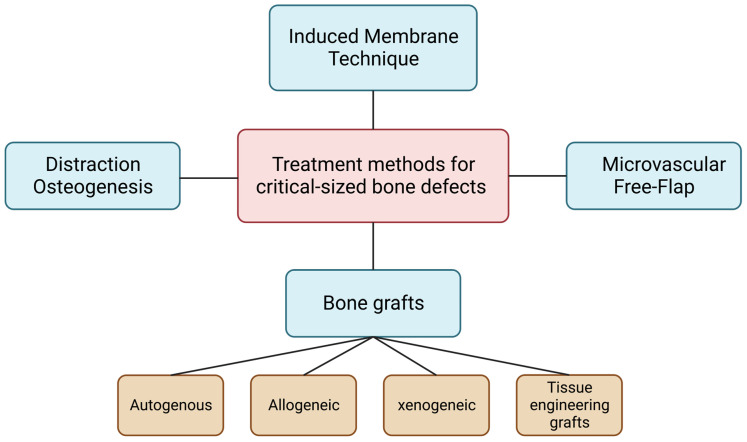
Treatment methods for critical-sized bone defects.

CSDs can be treated with different approaches, including bone grafts, microvascular free flap surgery, induced membrane technique, and distraction osteogenesis, as illustrated in Figure 1.

### 2.1. Bone Grafts

Bone grafting is a surgical procedure used to restore critical-sized osseous defects. Bone grafts act as a filler and scaffold to facilitate healing and new bone formation. The four types of bone grafts used for CSDs are autogenous, allogeneic, xenogeneic, and tissue engineering grafts [23].

#### 2.1.1. Autogenous Bone Grafts

Autogenous grafts are derived from one region of the patient’s body and transferred to another location. Bone can be harvested from intro-oral or extra-oral sources [23]. Autogenous grafts are known as the “gold standard” of bone grafts as they are widely used in clinical practice without eliciting immune responses [24]. Autogenous bone grafts have significant osteogenic, osteoinductive, and osteoconductive properties [23]. Studies have shown that autogenous bone gives the best post-bone grafting results [25]. However, disadvantages do exist due to the complications of additional surgical procedures [26].

#### 2.1.2. Allogeneic Bone Grafts

Allogeneic bone grafts are derived from cadavers and used on live patients’ bodies. The use of allograft requires sterilization and deactivation of proteins normally found in healthy bone [23]. Today, the demineralized freeze-dried bone allograft is widely used in regenerative procedures due to an increase in shelf-life and lower immunologic response as opposed to the fresh and frozen allograft. The use of allograft minimizes surgical time and reduces surgical complications. However, allogeneic bone grafts do pose the risk of rejection and transmission of disease if sterilization protocols are not followed [27].

#### 2.1.3. Xenogeneic

Xenogeneic bone grafts are derived from a donor species other than a human [23]. The material is removed from its organic components to prevent an immunologic response and is used as a calcified matrix [24]. Xenografts show osteoconductive properties and are chemically and physically similar to human bone [28]. However, like allogeneic bone grafts, there is a risk of rejection and transmission of disease if sterilization protocols are not followed [29].

#### 2.1.4. Tissue Engineering Bone Grafts

Tissue-engineered bone grafts can be a viable option to restore CSDs in the future [30] and alleviate the shortage of allograft, autograft, and xenogeneic bone grafts [31]. Tissue-engineered bone grafts would have the same repair and regeneration mechanisms as the native bone. This field is currently developing due to the advances in stem cell biology and biomaterials [30]. The application of such scaffold in enhancing vascularization and bone formation has been reviewed elsewhere [32,33].

### 2.2. Microvascular Free Flap

Microvascular free flap surgery is conducted for patients with large bone defects in the oral and maxillofacial region. Defects can occur due to tumor removal, massive trauma, or osteomyelitis. These defects can alter the region’s function and aesthetics, ultimately reducing the patient’s quality of life. A microvascular free flap can be a section of skin, tissue, muscle, and/or bone that is removed from one region and transferred to the recipient site. These sections can be taken from the fibula, radius, iliac crest, ribs, or scapula. The fibula is known to be the preferred donor site for mandibular reconstructions [34]. Clinical success is dependent on preoperative planning, technical skill, and post-operative monitoring [35]. However, complications can arise due to invasive surgical procedures and microvascular anastomoses.

### 2.3. Induced Membrane Technique

The induced membrane technique, also known as the Masquelet technique, is used to correct large skeletal deformities [36]. There are two phases of surgical treatment. During the first stage, a temporary PMMA cement spacer is placed into the defect. After a few weeks, the cement spacer is enclosed by an induced membrane, and a bone graft is placed into the cavity [37]. Although this technique is reliable and simple, determining the source of bone graft and debridement scope remains a challenge [36].

### 2.4. Distraction Osteogenesis

Distraction osteogenesis is a surgical technique of bone regeneration via lengthening [38,39]. Specifically, two segments of bone are gradually separated, and new bone is regenerated in the between gap [40]. Distraction osteogenesis is used in cases where guided bone regeneration is limited and where >5 mm of vertical bone height is required. This technique can be used to correct defects up to 15 mm and concurrently allows for soft tissue growth.

However, not only is it expensive and limited to vertical defects, but also its success depends on patient compliance [38]

## 3. Importance and Methods of Vascularization in CSDs

The complex biological healing of bone defects is directly controlled by vascularization (growing blood vessels into the bone tissue) or angiogenesis factors [41]. This is due to the fact that growth, differentiation, and tissue functionality directly depend on not only providing sufficient oxygen and nutrients but also removing waste products from the site with blood fellow [42]. Moreover, the lack of blood flow within a tissue can result in delayed healing, hypoxic cell death, ischemia, and ultimate failure in treatment [43,44,45]. The interdependency of angiogenesis and osteogenesis can be proved by the presence of osteoblasts and bone progenitor cells near vascular endothelial cells at the bone formation site [46].

In non-critical-sized defects, bone tissue represents a high regenerative ability. This successful bone regeneration in small defects is due to sufficient nutritional support and recruitment of bone progenitor cells. However, when it comes to CSDs, bone regeneration fails due to vascular system damage and limitation in the recruitment of bone-forming cells and nutrients [47,48]. This absence of vascularization is the reason for non-union and delayed-union defect development [49]. Non-union is defined as the nonhealing defect in which bone tissue is not produced. In contrast, in a delayed union, the bone production process is far slower than usual [49,50].

**Figure 2:**
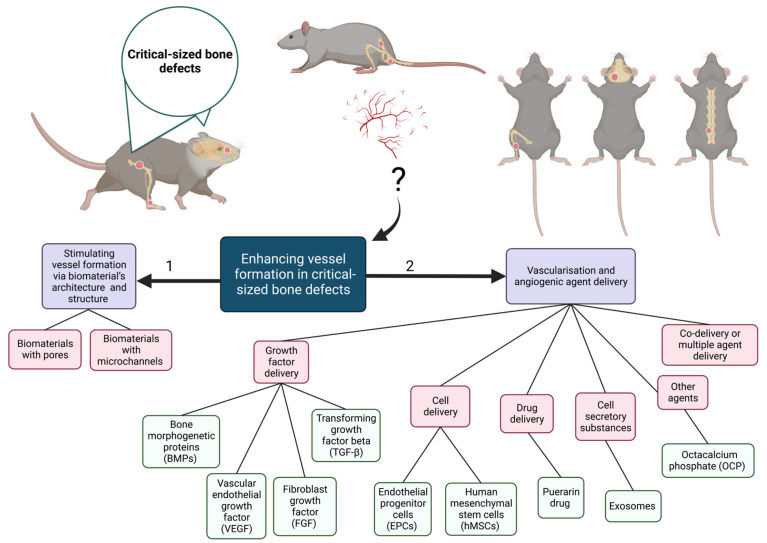
Enhancement methods for vascularization and angiogenesis in critical-sized bone defects via (1) Biomaterial’s architecture and structure or (2) Delivering angiogenic factors.

Various strategies have been developed to resolve poor vascularization challenges in tissue engineering. As illustrated in Figure 2, these methods can be classified into two main groups, including (1) application of biomaterials with the requisite properties and (2) growth factor, cells, particle, and drug delivery [22].

Biomaterials, such as scaffolds with microchannels or pores, can be designed to accelerate vascularization in tissues only with biomaterial architecture [51,52,53]. These biomaterials show promising results since the inner surface of the microchannels or pores controls cell recruitment and hence can be covered by endothelial cells [51]. For example, an alginate/gelatin hydrogel-based scaffold containing interconnected microchannels is designed using the 3D-printing method. According to the in vivo study, it has been shown that vessel formation has been facilitated in the center of the porous scaffold [54]. However, research on the effectiveness of these agent-free biomaterials in the vascularization of critical-sized bone defects is limited.

Another method to encourage vessel formation in CSDs, as can be seen in Figure 2, is delivering vascularization and angiogenic agents [22], such as growth factors (GFs), cells, drugs, other cell secretion substances agents, and even their combination, to the defected area [55,56,57,58]. These agents can be delivered to the site of injury via different methods, such as injection or incorporation into the biomaterials’ network or coating.

Here, the studies that have been performed regarding the effect of these agents on the vascularization of CSDs will be reviewed in separate sections. Moreover, the effect of vascularization on subsequent bone formation is evaluated for each agent.

### 3.1. Growth Factor Delivery for Vascularization of CSDs

The blood vessel formation can be stimulated by a variety of signal proteins, known as (GFs) [59]. The vascular endothelial growth factor (VEGF) superfamily plays the most important role in the vascularization and angiogenesis of tissues [60]. In addition to VEGF as the main angiogenic agent, a variety of other GFs can also improve vessel formation in bone tissue. Transforming growth factor beta (TGF-β), bone morphogenetic proteins (BMPs), and fibroblast growth factor (FGF) are examples of these successful agents [61,62,63]. Here, the effect of growth factor (GF) delivery on the vascularization of CSD will be reviewed comprehensively.

#### 3.1.1. VEGF Delivery for Vascularization of CSD

**Figure 3:**
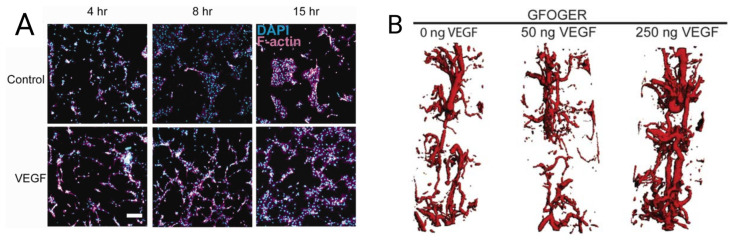
Vascularization of PEGylated VEGF. (**A**) Endothelial cell networks image on GFOGER/PEG with and without VEGF, (**B**) 3D reconstructions of vascular structure for different doses of VEGF. Adapted from [65] with permission by the corresponding author (2022).

VEGF, as a potent angiogenic factor, is produced by many cells to promote the formation of new blood vessels [64]. The effect of VEGF on vessel formation of murine critical-sized radial bone defects has been proved when covalently incorporated into poly (ethylene glycol) (PEG) hydrogel functionalized with triple-helical GFOGER peptide. The VEGF-loaded GFOGER-functionalized PEG showed higher and denser endothelial cell networks (Figure 3A,B) and endothelial nuclei count in comparison to the VEGF-free GFOGER/PEG hydrogel [65].

**Figure 4:**
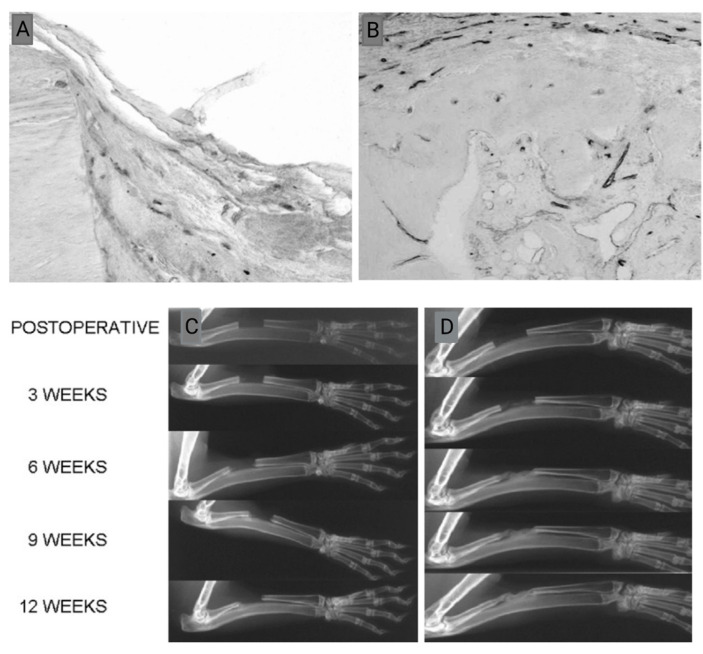
Comparison of the effect of VEFG 165 on vascularization and bone formation of CSD. (**A**) Vascularization of CSD in the control group without VEGF, (**B**) vascularization of CSD with VEGF (dark lines are representative of the newly formed blood vessel), (**C**) bone formation in the control group without VEGF 165 (atrophic non-union), (**D**) bone formation and in VEGF 165-treated group (bridging of the bone gap after 6 weeks). Adopted from reference [66] with permission by the corresponding author (2022).

In another study, it was proved that VEGF_165_ could promote angiogenesis and bone healing (Figure 4) in rabbit radial CSD (15 mm). When the defect was treated in the presence of VEGF, the number of newly formed vessels (dark lines in Figure 4B) was two or three times more than the control group (Figure 4A), and larger endothelial areas were formed [66].

**Figure 5:**
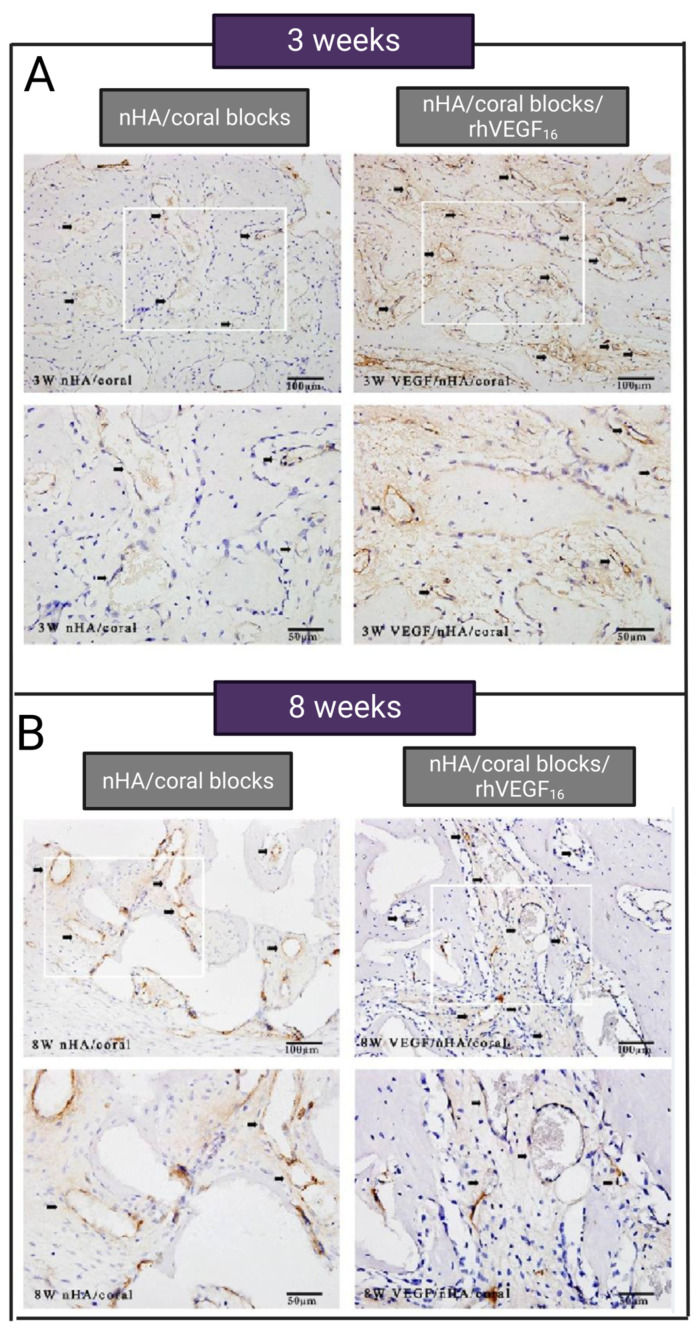
The comparison of the vessel formation in decalcified tissues of the control group treated with nHA/coral blocks and VEGF group treated with nHA/coral blocks/rhVEGF_165_, (**A**) 3 weeks after implantation, and (**B**) 8 weeks after implantation. Circular and brown vessels are shown with black arrows. Adopted from reference [67] with permission by the corresponding author (2022).

Vessel formation of CSD could also be promoted via VEGF_165_ when incorporated into the coralline hydroxyapatite (CHA) composite scaffold. Du et al. [67] coated nano-hydroxyapatite/coralline (nHA/coral) block grafts with recombinant human vascular endothelial growth factor_165_ (rhVEGF). It has been proven that neovascularization is improved when nHA/coral blocks/rhVEGF_165_ scaffold was applied to treat CSD in dogs’ buccal bone (9 × 6 × 12 mm) [67]. As illustrated in Figure 5A,B, more blood vessels (shown via black arrows) are formed in the VEGF group 3 and 8 weeks after implantation [67]. The blood vessel density in the control and VEGF-treated group after 3 weeks is calculated at 105 ± 51.8 and 146 ± 32.9 vessel/mm^2^, respectively. A total of 8 weeks after implantation, the neovascular densities of both groups had significantly increased to 269 ± 50.7 and 341 ± 86.1 vessel/mm^2^ in the control and VEGF-treated groups, respectively [67].

#### 3.1.2. TGF-β Delivery for Vascularization of CSD

The TGF-β superfamily, as multipotent GFs, participates in a variety of cellular processes and hence affects cell proliferation, differentiation, and apoptosis [68]. The largest reservoir of TGF-β is bone tissue since it is produced by osteoblasts and is stored in the bone matrix [69]. At an early stage in fractures, TGF-β1 is found in the periosteum (a membrane that covers the outer surface of all bones) and increases the formation of microvessel-like structures [70], and enhances the proliferation of osteoblasts and mesenchymal cells in fractures [71]. However, the TGF-β superfamily can play the role of an angiogenic and angiostatic factor on vein endothelial cells (VECs) depending on tissue context, level of expression, and interaction with other mediators [72]. Hence, they can be used in the vascularization of CSDs.

For instance, in the rat critical-sized calvarial defects (8 mm diameter), growth differentiation factor 15 (GDF15), as a divergent member of the TGF-β superfamily, could promote angiogenesis and the proliferation of human umbilical VECs [73]. In this study, first, β-tricalcium phosphate (β-TCP) scaffolds containing bone marrow mesenchymal stem cells (BMSCs/β-TCP) were prepared. Next, rats were divided into five groups and received different treatments for the critical-sized calvarial defect. Rats in groups A, B, and C received BMSCs/β-TCP implants with local administration of GDF15, GDF15/Bevacizumab, and antibodies to GDF15, respectively. Groups D and E were treated with BMSCs/β-TCP and β-TCP implant only [73]. In this study, bevacizumab was used as it prevents the VEGF binding to the receptor of the cell surface and hence plays the role of VEGF inhibitor [74].

**Figure 6:**
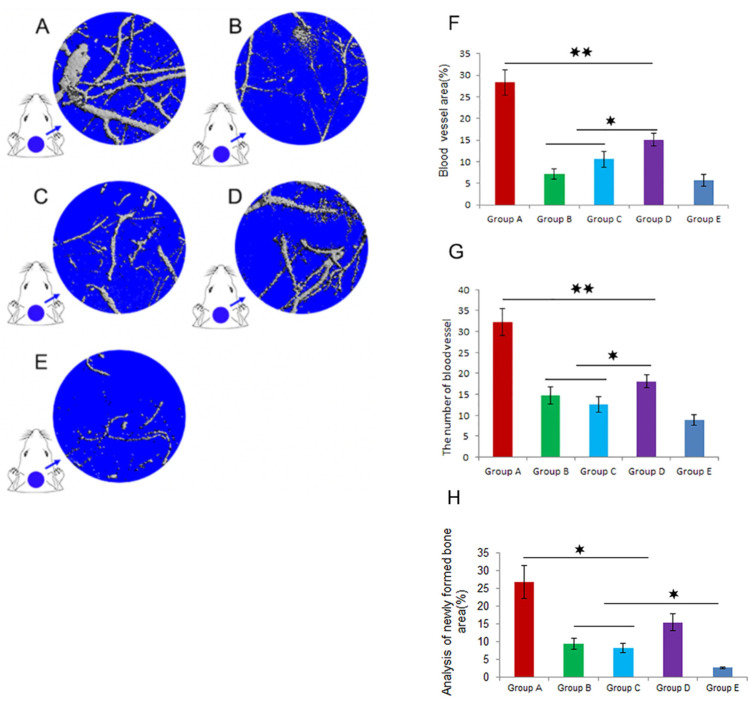
The effect of different treatments on angiogenesis and bone formation of calvarial CSDs at 12 weeks post-surgery. (**A**–**E**) Comparison of blood vessel formation by microfilm perfusing. (**F**) blood vessel area, (**G**) the number of the blood vessel, (**H**) newly formed bone (* *p* < 0.05 and ** *p* < 0.01). Adopted from reference [73] with permission by the corresponding author (2022).

Figure 6A–E represents microfilm perfusion results using micro-CT measurements for each treatment. A total of 12 weeks after implantation, group A (Figure 6A), which received GDF15 with BMSCs/β-TCP scaffold, showed the highest blood vessel area and blood vessel number, as illustrated in Figure 6F,G, respectively. Moreover, Groups B and C showed the lowest vascularization since they received VEGF inhibiting agents (Bevacizumab and antibody to GDF15). Figure 6H proves the close association of osteogenesis with angiogenesis. The higher the blood vessel area and the number of vessels, the higher the amount of newly formed bone. Hence, two hypotheses have been proved in this study: (1) the positive effect of GDF15 in increasing the angiogenesis and vascularization of critical-sized bone defects and (2) the positive effect of vessel formation on bone generation [73].

#### 3.1.3. BMP Delivery for Vascularization of CSD

BMPs are also another group of bone GFs; they change undifferentiated mesenchymal cells into osteoblasts [75] and provoke and induce bone formation heterotopically [76,77]. However, in a study, it has been proved that aside from bone formation, BMP-2 can facilitate vascularization in rabbits’ critical-sized radius defect (GS) [55]. In this study, radius CSD in rabbits was divided into four groups and treated with: (1) empty gelatin sponge (G), (2) gelatin loaded with BMP-2 (BMP-2/G), (3) gelatin loaded with a mixture of BMP-2 and 2-N,6-O-sulfated chitosan (BMP-2/26SCS/G), and (4) gelatin loaded with BMP-2 containing SC nanoparticles (BMP-2/S-NP/G) [55].

**Figure 7:**
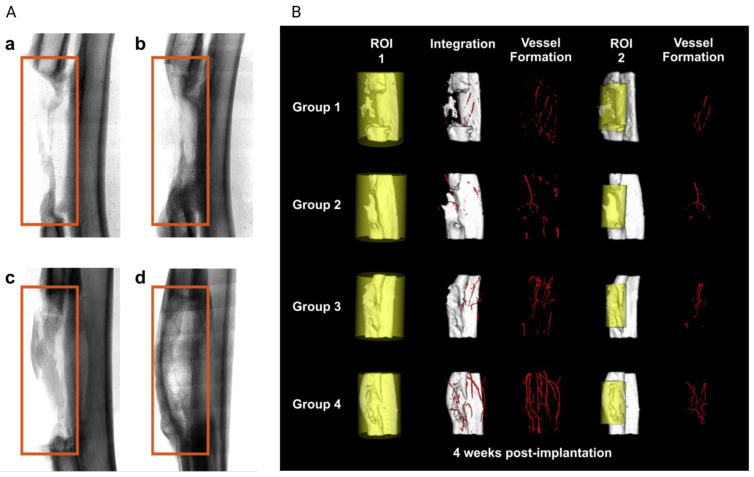
Comparison of (**A**) bone regeneration 2 weeks after implantation (a: group 1, b: group 2, c: group 3, d: group 4), (**B**) bone regeneration and vessel formation 4 weeks after implantation, adopted from reference [55] with permission by the corresponding author (2022).

As depicted in Figure 7A, 2 weeks after implantation, bone mineral content was slightly higher in group 2 and significantly higher in groups 3 and 4 compared to group 1 (empty sponge). Moreover, the connective bridge was observed between two ends of the defect in group 4 (sponge containing BMP-2 loaded SC nanoparticles).

The bone and vessel formation (in two regions of interest (ROI1 and ROI2)) were studied via μCT imaging and microangiography (Figure 7B). According to the results, 4 weeks post-implantation, the slowest bone and vein formation was attributed to group 1 (empty sponge). In contrast, group 2 (sponge with BMP-2) showed better integrity and repair rate and slightly better vessel formation. In group 3, although at week 4, suitable trabecular bridging and higher vessel volume were observed, there was still a large defect. The highest bone formation and richest peripheral vessels were attributed to group 4, where the gelatin sponge contains BMP-2-loaded SC nanoparticles [55]. This higher vascularization and bone formation in group 4 compared with group 3 can be due to the sustainable BMP-2 release with lower initial burst release from the SC nanoparticles [55].

#### 3.1.4. FGF Delivery for Vascularization of CSD

The FGF superfamily is synthesized by osteoblasts, chondrocytes, monocytes, and macrophages [78], and in bone tissue, they regulate cell functions such as differentiation, proliferation, and migration. It has been proven that significant neovascularization enhancement in bone tissue can be caused by the application of bFGF [79].

It has been shown that bFGF is capable of increasing VEGF expression in CSDs [80]. For instance, Baker et al. [80] proved that the presence of bFGF in rabbit mandibles CSD bilaterally (15 mm × 10 mm) not only can increase the expression of VEGF but also enhance allogeneic bone healing. Hence, aside from the effectiveness of bFGF in the vascularization of CSD, a close and direct correlation is shown between enhanced vascularization and bone formation in vivo [80].

#### 3.1.5. Dual or Combined Growth Factor Delivery for Vascularization of CSD

**Figure 8:**
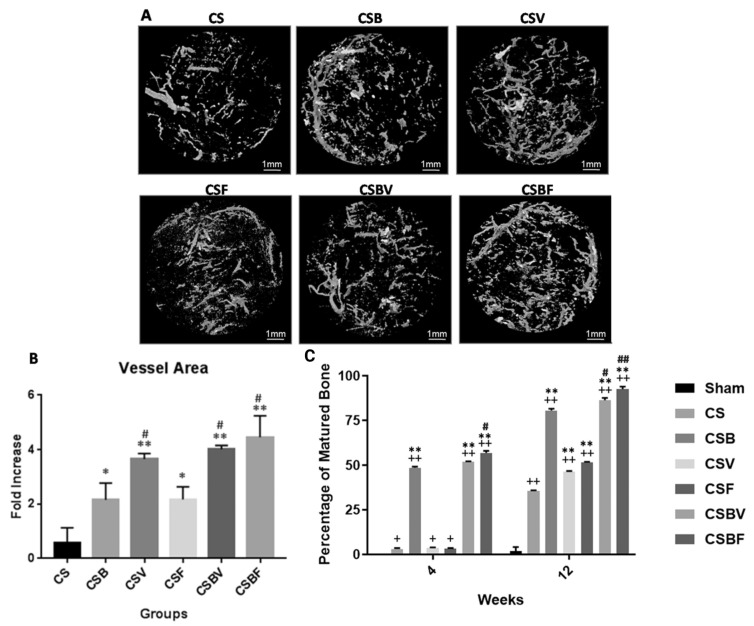
Comparison of the (**A**) vascularization through the nanocomposite fibrous scaffold implanted in CSD in different groups 4 weeks after implantation: (**B**) vessel area 4 weeks after implantation (*—*p* < 0.05, **—*p* < 0.001, #—*p* < 0.05); (**C**) mature bone formation 4 and 8 weeks after implantation (+—*p* < 0.05, ++—*p* < 0.001, **—*p* < 0.001, #—*p* < 0.05, ##—*p* < 0.001). Adopted from reference [81] with permission by the corresponding author (2022).

Dual or combined release of GFs can stimulate vascularization synergistically in CSD. Kuttapan et al. [81] studied the synergistic effect of combining two GFs on vessel and bone formation in rat critical-sized calvarial defect (8 mm diameter and 1.5 mm thickness). In this study, rats were divided into six groups and received (1) CS: nanocomposite fibrous scaffold, (2) CSB: scaffold + BMP2, (3) CSV: scaffold + VEGF, (4) CSF: scaffold + FGF2, (5) CSBV: scaffold + BMP2 + VEGF, and (6) CSBF: scaffold + BMP2 + FGF2. As can be seen in Figure 8A,B, in comparison to group 1 (received bare scaffold), a higher level of vascularization can be observed for all groups treated with either single or dual systems [81]. In general, the highest vascularization is attributed to CSBV and CSBF as dual systems and the CSV single system. Although the scaffold with VEGF (single system) showed an acceptable vascularization, it was not successful in bone regeneration 4 and 12 weeks after implantation (Figure 8C), whereas BMP2 administration in single and dual systems had a positive effect on both vessel and bone formation 4 and 12 weeks after implantation [81]. This study approves the synergetic effect of dual GF delivery on the vascularization and osteogenesis of CSD. However, the correlation between higher blood vessel formation and better bone formation was disapproved in this study since CSV with high vascularization (Figure 8B) did not show high bone formation (Figure 8C) [81].

**Figure 9:**
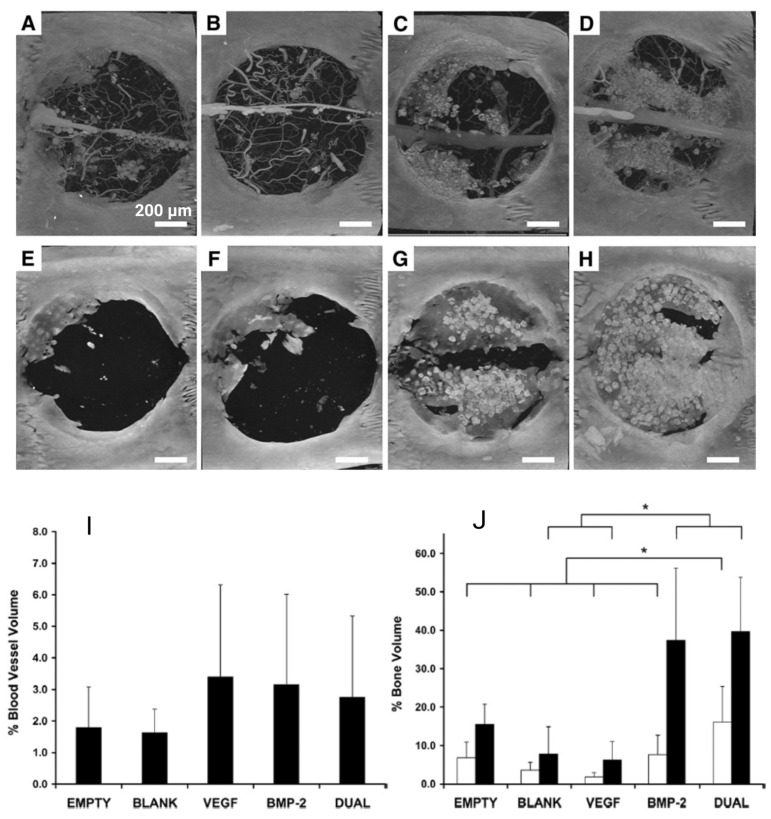
Comparison of the vessel and bone formation in 4 groups at 4 and 12 weeks post-implantation. (**A**–**H**) Cranial defects micro-CT images at 4 and 12 weeks. (**A**) BLANK, (**B**) VEGF, (**C**) BMP-2, and (**D**) DUAL groups at 4 weeks. In (**A**–**H**) panels, both blood vessels and bone are visible. (**E**) BLANK, (**F**) VEGF, (**G**) BMP-2, and (**H**) DUAL groups at 12 weeks. In (**E**–**H**) panels, microfilm perfusion is not done, and blood vessels are not visible. (**I**) blood vessel volume quantification at 4 weeks post-implantation, (**J**) bone volume quantification at 4 and 12 weeks post-implantation, (*—*p* < 0.05). Adopted from reference [56] with permission by the corresponding author (2022).

This hypothesis was disproved in a study conducted by Patel et al. [56] when the effect of dual delivery of angiogenic and osteogenic GFs was studied in CSD treatment [56]. In this study, rat cranial CSDs (8 mm diameter) were treated with a porous scaffold incorporated with gelatin microparticles via (A) no GF (blank group), (B) VEGF, (C) BMP-2, and (D) both VEGF and BMP-2 [56]. Figure 9A–H represents the micro-CT images of cranial CSDs 4 and 12 weeks after implantation. Figure 9I,J represent the comparison of vessel and bone formation, respectively. The following conclusions can be drawn from the mentioned figures. First, no significant difference was observed in blood vessel volume among groups (Figure 9I). Although the VEGF group showed slightly better vessel formation 4 weeks after implantation (Figure 9I), this GF had no impact on bone formation 4 and 12 weeks after implantation (Figure 9J) [56]. Second, although higher bone formation is observed in BMP-2 and dual (BMP-2 and VEGF) groups, the bony bridging was observed in animals treated with dual release [56].

Hence, the synergistic effect of the dual delivery of VEGF and BMP-2 on bone formation has been proven in this study, but no relationship was found between the formation of more blood vessels and better bone regeneration in a CSD model. This phenomenon can be explained by the immaturity of the formed blood vessel network in the presence of VEGF only that can be trimmed or remodeled [82]. These results are in contrast with the previous studies that demonstrated the effect of better vessel formation on higher bone generation via growth factor delivery in CSD [83]. This contradistinction can be due to two reasons. The first reason is related to the differences in GF release kinetics, delivery vehicle, implantation site, animal model, and VEGF dose, which can cause such contradiction [56]. The second reason, as can be seen in Figure 9I,N results, is the high standard deviation, which needs further research. Hence, such a high standard deviation may be the cause of this contradiction as well.

Another study that disproved the correlation between better vessel formation and higher bone formation via GF delivery in CSDs was conducted by Lv et al. In this study, porous titanium scaffolds were impregnated via GF-doped fibrin glue and used to treat rabbit medial femoral condyle CSDs (5 mm diameter by 6 mm depth) [10]. Fibrin glue played the role of carrier material for the controlled and sustained release of GF through porous scaffolds to improve the bioactivity of an implant. Rabbits were divided into five study groups and received: (1) empty scaffolds, (2) scaffold containing undoped fibrin glue, (3) scaffold impregnated with fibrin glue containing BMP, (4) scaffold impregnated with fibrin glue containing VEGF, and (5) scaffold impregnated with fibrin glue containing rhBMP-2 and VEGF [10].

Three conclusions can be drawn from the results: First, both angiogenesis and osteogenesis inside the porous scaffolds are enhanced by the application of a single GF or dual GFs. Second, dual factors combination showed a synergistic effect on angiogenesis while this synergy was absent on osteogenesis. Third, higher blood vessel formation does not provide better bone formation [10].

Aside from the disapproval of the correlation between vessel and bone formation, the synergistic effect of the dual delivery of VEGF and BMP-2 on bone formation was also disproved in this study. These findings are in contrast to previous evaluations [56]. This contrast can also be explained by differences in the animal model (rabbit and rat), delivery vehicle (fibrin glue and gelatin microparticles), implantation site (medial femoral condyle and cranial), and growth factor dose.

Another reason for such contradiction can be the type of vessel that is newly formed in CSDs. Two subtypes of vessels exist in the bone marrow cavity and capillaries of the metaphysis [84]. These types are known as type H and type L vessels [84,85]. It has been proven that a large number of bone progenitor cells are distributed around type H vessels [86]. In contrast, no surrounding bone progenitor cells have been found around type L vessels [84]. These progenitors can accelerate the bone formation and increase bone mass by differentiating into osteoblasts and osteocytes [84,85]. Hence, bone formation in CSDs can depend on the existence and number of H-type vessels in the newly formed vascular network.

### 3.2. Cells Delivery for Vascularization of CSDs

Apart from growth factor delivery, cell delivery can also encourage the formation of new blood vessels. For example, delivering endothelial progenitor cells (EPCs) [87], human umbilical vein endothelial cells [88], and human mesenchymal stem cells (hMSCs) [58,87] to the site of injury showed promising results in enhancing CSDs’ vascularization. EPCs are known as the precursor cells of vascular endothelial cells (VECs) that can be used to form mature blood vessels [89,90]. hMSCs, due to their high potential in differentiating from the VECs in bone engineering applications, showed great potential in improving angiogenesis with the formation of umbilical veins [91]. For example, adipose-derived mesenchymal stem cells (AdMSCs) can improve angiogenesis not only by differentiating into VECs but also through the secretion of VEGF [92,93,94]. Moreover, it has been suggested that traditional single-cell delivery should be replaced by co-culture cell systems as they can synergistically promote both vascularization and vascularized bone formation.

The effect of both single-seed cells and co-culture systems on vascularization, angiogenesis, and bone formation of CSDs has been studied by Seebach et al. [87]. The femoral CSDs in adult athymic rat groups were divided into five groups and filled with fibronectin-coated β-TCP granules, EPCs seeded on β-TCP, MSCs seeded on β-TCP, co-culture of MSC + EPC seeded on β-TCP, and autologous bone graft [87]. According to the results, five conclusions can be drawn from this study. First, after 1 and 4 weeks, the formation of a primitive vascular plexus was increased in rats receiving EPCs in comparison with the MSC group. However, 8 weeks after implantation, the MSC-receiving group demonstrated a higher degree of vascularization compared to the EPC group [87]. Second, after 1, 4, and 8 weeks, scaffolds receiving the co-culture system (β-TCP/EPCs + MSCs) demonstrated a significantly higher degree of vascularization in comparison to other samples. Third, the co-culture group showed the highest bone formation at weeks 4 and 8. Fourth, although at week 8 both β-TCP + MSCs (single-cell seed) and β-TCP + EPCs + MSCs (co-culture system) showed a high area of vascularization, the area of bone formation was higher in the co-culture-receiving group. Hence, not only the existence of a close relationship between blood vessels formation and bone development is confirmed in this study, but it has also been proven that dual delivery of EPCs and MSCs synergistically promotes vascularized bone formation compared to single delivery of EPCs or MSCs [87].

**Figure 10:**
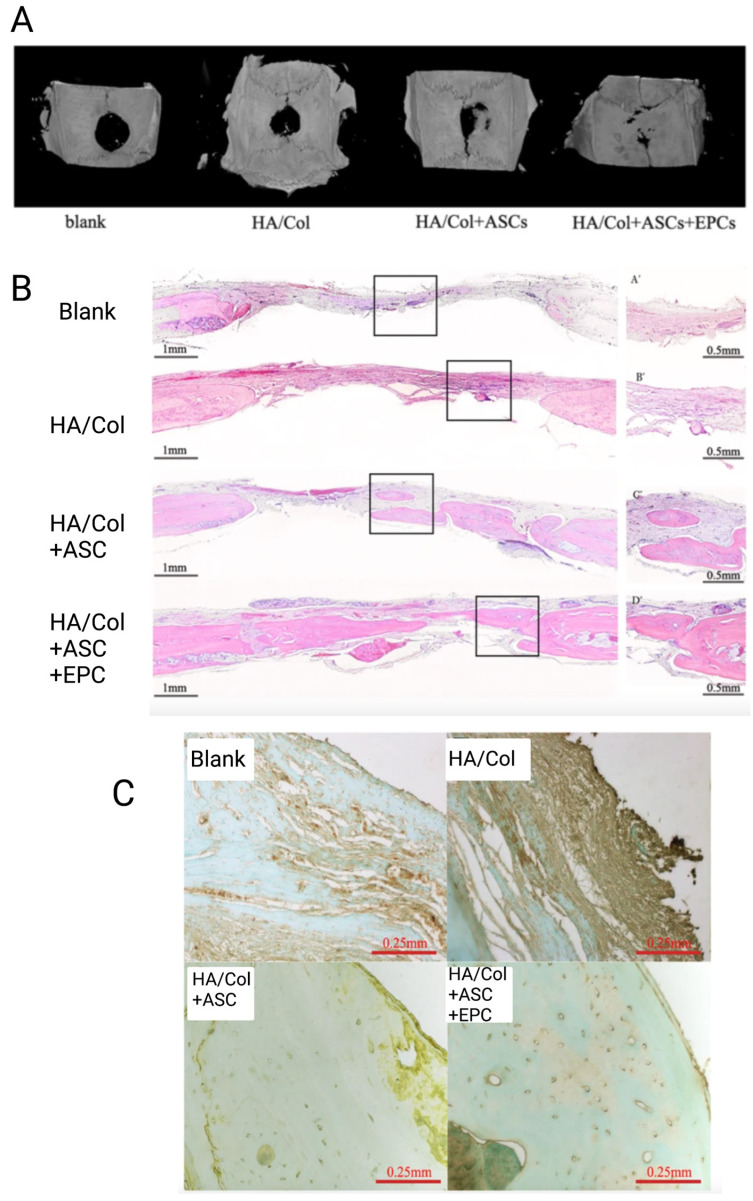
Bone and vessel formation comparison between different groups 8 weeks after surgery, (**A**) the Micro-CT scan images of repaired cranial bones, (**B**) new bone formation, (**C**) VEGF immunostaining in the newly formed bone. Adpted from reference [48] with permission by the corresponding author (2022).

In another study, the effect of EPCs and AdMSCs co-culture system on vascularization and repairing of critical-sized cranial bone defects in rats was studied and compared with the effect of single-cell delivery (AdMSCs) [48]. In this study, a hydroxyapatite/collagen (HA/Col) scaffold was prepared to be combined with a single or co-culture cell system. Next, rats were classified into four groups according to the treatment they received, including (1) blank group (did not receive any treatment), (2) HA/Col scaffold group (just received scaffold material), (3) HA/Col + AdMSCs group (single-seed cells), and (4) HA/Col + AdMSCs + EPCs group (co-culture cell system) [48]. He et al. [48] confirmed that the implants treated with co-culture of AdMSCs and EPCs show dramatically higher bone density (Figure 10A), bone area (Figure 10B), and vessel formation (Figure 10C) in comparison to other groups. Moreover, it is confirmed that single-cell groups showed higher bone and vessel formation compared to blank or scaffold groups. These results are in accordance with findings reported by Seebach et al. [87] and prove the same two points. First, the exitance of a close relationship between blood vessel formation and bone development; Second, the higher and synergetic effect of co-culture cell systems on vascularization and angiogenesis in comparison to single-seed cells [48]. However, it is worth mentioning that these results need further investigation due to the absence of a HA/Col + EPCs (single-seed cell) study group in this research.

Although cell delivery has become a promising approach for increasing the vascularization of CSDs, the major limitations of this therapy are the death and apoptosis of implanted cells due to hypoxia in the severe condition of CSDs [95]. To be more specific, cells are cultured in normoxic conditions (21% O_2_). In contrast, in the bone defect area, ischemia and hypoxia (<1% O_2_) remained a problem. Therefore, increasing the survival rate of the transplanted cells will improve angiogenesis and vascularization. One solution to this problem is the application of hypoxia-inducible factor (HIF)-1. This factor is capable of responding to low oxygen concentrations by providing oxygen-independent adenosine triphosphate (ATP) production and inhibits cell apoptosis [95].

Aside from increasing cell viability and survival, HIF-1α can activate angiogenic genes such as VEGF, bFGF, TGF-β, placental growth factor (PLGF), angiopoietin 1 (ANGPT1), and stem cell factor (SCF) [96,97,98]. Hif-1α can bind to HIF-1β after stabilizing and accumulation and form the HIF-1 complex. These complexes stimulate VEGF expression and consequently improve neovascularization [99,100].

HIF-1α can be transduced into cells by lentivirus vectors. Lentiviral vectors had success in clinical gene therapy applications. These lentiviruses attach to the surface of target cells and inject viral material into the cytoplasm [101]. Delivering HIF-1α transgenic BMSCs could increase the number and volume of blood vessels in osteonecrosis of the femoral head [102].

**Figure 11:**
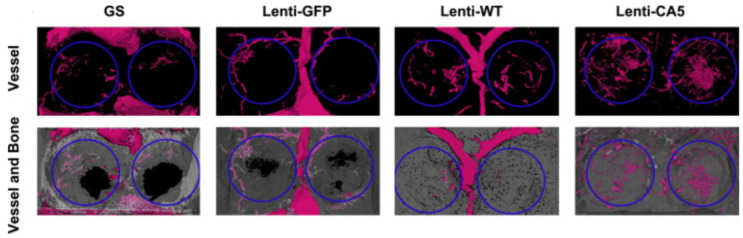
Comparison of the effect of HIF-1a gene therapy on BMSCs vascularization in tissue-engineered bone via reconstructions of the three-dimensional micro-CT images 8 weeks after implantation [103]. Adopted from reference [103] with permission by the corresponding author (2022).

To enhance angiogenesis in CSDs, using a lentivirus vector, BMSCs were treated with three factors: (1) enhanced green fluorescent protein (Lenti-GFP), wild-type of HIF-1α (Lenti-WT), and constitutively active form of HIF-1α (Lenti-CA5) [103]. Next, rats with calvaria CSDs (5 mm diameter) were divided into the four following groups and received treatments as follows. Group I received gelatin sponge scaffold (GS), group II was treated with GS containing BMSCs/Lenti-GFP, group III received GS containing BMSCs/Lenti-WT, and group IV received GS containing BMSCs/Lenti-CA5 [103]. As has been illustrated in Figure 11, both the Lenti-WT and Lenti-CA5 groups showed significantly higher vessel and bone formation 8 weeks after implantation, in comparison to the Lenti-GFP and GS groups. Moreover, the CA5 group represents a more dense vascular network and bone mineral density [103]. Hence, one effective method to increase angiogenesis in CSDs is HIF-1α gene therapy on BMSCs and delivering these cells to the site of injury.

**Figure 12:**
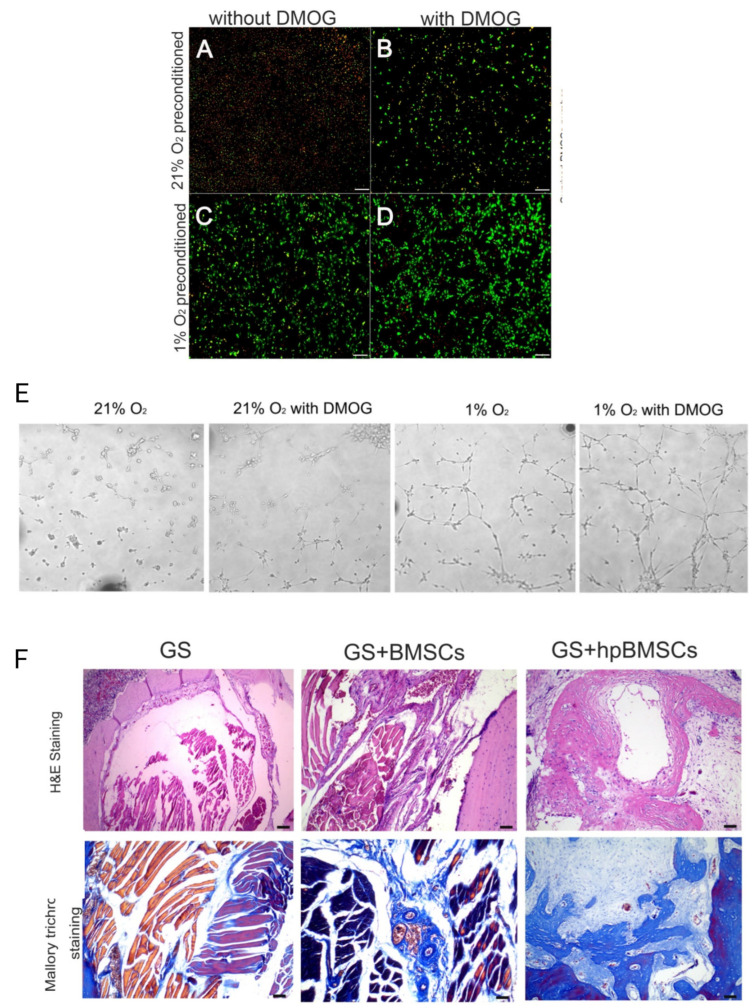
The effect of DMOG on cell viability, HIF-1α tube, bone, and vessel formation. (**A**–**D**) Comparison of BMSCs survival with and without DMOG in hypoxic or normoxic conditions. (**E**) Comparison of angiogenic activities of BMSC with and without DMOG in hypoxic or normoxic conditions. The highest tube formation is attributed to hypoxia-preconditioned cells with DMOG and 1% O2. (**F**) Histological analysis of bone defect area. Adopted from reference [9], with permission by the corresponding author (2022).

However, the HIF-1α proteins degrade rapidly. Hence, the survival rate of the transplanted cells and angiogenesis can be enhanced by up-regulating the protein expression level of HIF-1α. One of the methods to up-regulate the expression level of this protein under normoxic conditions is the application of dimethyloxaloylglycine (DMOG) as a cell-permeable prolyl-4-hydroxylase inhibitor [104]. For example, it has been reported that DMOG could improve the survival rate of cells, the angiogenic activity of BMSCs, mRNA expression, and the secretion of related angiogenic factors by activating the expression of HIF-1α [104,105]. The angiogenesis and osteogenesis of rats’ critical-sized mandible defects could also be improved when treated with hypoxia-preconditioned BMSCs (with 0.5 mM DMOG and 1% O_2_) [9]. Zahng et al. [9] treated BMSCs with 0.5 mM DMOG and 1% O_2_ to achieve hypoxia-preconditioned BMSCs for CSD angiogenesis. In this study, the survival rate of BMSCs in hypoxic or normoxic condition with and without DMOG is compared. It has been proven that the highest survival rate and HIF-1α expression level are attributed to hypoxia-preconditioned BMSCs with 0.5 mM DMOG and 1% O_2_ (Figure 12A–D). Moreover, when BMSCs were cultured in hypoxic conditions with DMOG, they formed more tubes in comparison to those cultured in normoxic conditions or without DMOG (Figure 12E) [9]. Next, critical-sized mandible defects in rats (5 mm diameter) were treated with a gelatin sponge (GS), GS with regular BMSCs, and GS with hypoxia-preconditioned BMSCs (hpBMSCs). A total of 12 weeks after implantation, more blood vessels and bone area were observed in rats when treated with GS/hpBMSCs, as illustrated in Figure 12F [9]. Hence, in cell therapy techniques for the vascularization and angiogenesis of CSDs, DMOG can not only increase the cell survival rate after transplantation but also can increase the cell’s both angiogenesis and osteogenesis activity [9].

### 3.3. Vascularization of CSDs with Other Agents (Drugs and Cell Secretory Material)

**Figure 13:**
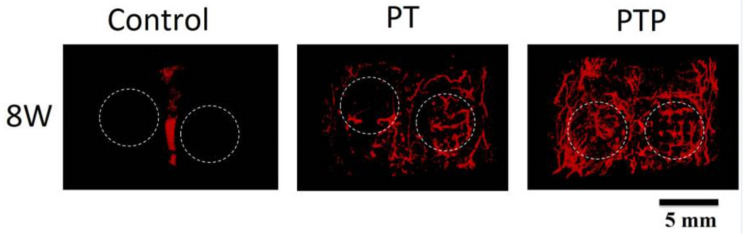
Three-dimensional reconstructed images of micro-CT-based angiography to compare vascularization in control, PT, and PTP groups at 8 weeks post-implantation in the cranial CSD. Margins of original defect are represented by dashed white lines. Adopted from reference [8], with permission by the corresponding author (2022).

In addition to Gf and cell delivery, other agents such as drugs and cell secretory substances can also stimulate vessel formation in CSDs [7,8]. For example, the incorporation of puerarin drug into the PT scaffold (PLGA/TCP, (poly (lactic-co-glycolic acid)/β-TCP)) showed promising results in repairing rat calvarial CSD (5 mm diameter and 1 mm thickness) via synergistic angiogenic and osteogenic effects [8]. Porous PLGA/TCP or PT scaffold has been proven to have suitable osteoconductive activity and biocompatibility. Puerarin, as an antioxidant drug, showed osteogenesis ability [106,107,108]. However, as illustrated in Figure 13, when puerarin is incorporated into the PT scaffold (PTP), it could promote vessel formation in the defected area. Moreover, it has been reported that PTP scaffold, by the stable release of puerarin, could stimulate osteogenesis as well. Osteogenesis enhancement occurs via three mechanisms. First, the formed vascular network can mediate the interaction of the surrounding cells by their recruitment. Second, puerarin combines with Ca^2+^ ions that are released from the scaffold and participate in bone repair. Third, puerarin can facilitate osteoblastic differentiation and mineralization [8].

**Figure 14:**
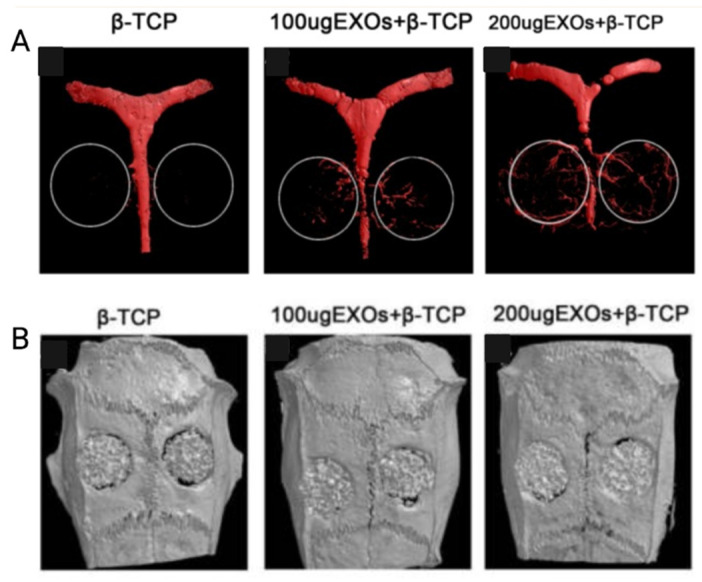
Comparison of (**A**) angiogenesis and (**B**) bone formation 8 weeks after implantation in three groups of rats treated with β-TCP alone, β-TCP containing 100 µg/mL, and β-TCP containing 200 µg/mL exosome. Adopted from reference [7], with permission by the corresponding author (2022).

Cellular secretion substances can also play the role of angiogenic drugs that can be used to enhance vessel formations in CSDs. For example, it has been illustrated that exosomes secreted by MSCs can dramatically stimulate angiogenesis and bone formation in rat calvarial CSDs (5 mm diameter) in a dose-dependent manner [7]. This effect is proven when β-TCP scaffolds containing exosomes (β-TCP + 200 µg EXCOs and β-TCP + 100 µg EXOs) were used to treat rats’ CSDs and compared with β-TCP alone [7]. β-TCP scaffolds containing exosome markedly promotes both angiogenesis (Figure 14A) and bone regeneration (Figure 14B). This angiogenesis and osteogenesis promotion was higher in scaffolds containing higher doses of exosome [7].

Apart from drugs and cell secretory substances, materials such as octacalcium phosphate (OCP) can also improve CSD vascularization via releasing ionic agents [109]. OCP is capable of stimulating angiogenesis before bone formation in CSDs. This vessel formation is attributed to OCP’s ability to change the ionic diffusion environment, as comprehensively explained elsewhere [109,110,111,112]. However, when OCPs were incorporated into the gelatin composite and implanted into the rat’s calvarial CSD, it could increase the volume of the new blood vessel 2 and 4 weeks after implantation [109].

### 3.4. Vascularization of CSDs via Combined Delivery

Another promising strategy for enhancing vascularization in CSDs is the local delivery of multiple agents with different natures at the same time. For instance, transplantation of PDGF-incorporated AdMSCs spheroids (combination of GF and cell) into mouse calvarial CSD could not only increase the capillaries and arterioles numbers after two months but also significantly enhance regenerated bone area [113]. To develop complex vascularized structures in CSDs, Lee et al. incorporated AdMSCs with 50 ng PDGF (PMF50) and with bio-mineral-coated fibers (MF) separately. The following conclusions have been drawn from this study: First, the incorporation of PDGF with AdMSCs increases not only the proliferation of AdMSCs but also their endothelial and osteogenic differentiation. Second, when AdMSCs are transplanted with MF, the capillaries and arterioles numbers increase in the defected site in comparison to the control group, but this enhancement is less than the PMF50 group (PDGF + AdMSCs). Third, AdMSCs combined with PDGF can enhance the regeneration of bone area since the highest newly formed bone area and bone volume (BV)/total volume (TV) is calculated for the PMF50 group [113]. Hence, it has been proven that successful vascularized bone regeneration followed by enhanced vessel formation can be achieved with the combined delivery of eighter AdMSCs-PDGF (stem cell-GF combination) or AdMSCs-biominerals (stem cell-biominerals combination) [113].

Another example of combined delivery for the vascularization of CSDs is the combination of AdMSCs with Sr-HT-Gahnite biomaterial. Sr-HT-Gahnite (or calcium silicate-based porous strontium-hardystonite-Gahnite) contains zinc and strontium ions [114]. This bioceramic scaffold, in comparison to calcium-silica bioceramic, exhibits superior angiogenesis and osteoinductivity due to the Sr incorporation. AdMSCs, as previously reviewed in this literature, have high angiogenesis and osteoinductivity [115,116]. It has been illustrated that the combination of both elements (AdMSCs with Sr-HT-Gahnite biomaterial) can promote both osteogenesis and angiogenesis in in vivo studies [114]. Wang et al. [114] compared the vascularization and bone formation of rats’ calvarial CSDs in four groups receiving different treatments. The first group of rats received β-TCP/hydroxyapatite (TCP/HA) scaffold (control). The second group received Sr-HT-Gahnite. The third group received a combination of TCP/HA and AdMSCs, and the last group was treated with a combination of Sr-HT-Gahnite and AdMSCs. Vessel formation and bone area increased drastically when rats received the combination of Sr-HT-Gahnite and AdMSCs [114].

## 4. Conclusions

CSDs are defined as the smallest defects that cannot be healed without surgical intervention within a patient’s lifetime. To support the healing process, an appropriate vascular system can be vital. This is due to the fact that bone is a vascularized tissue, and its growth and functionality directly depend on providing sufficient oxygen and nutrients and removing waste products from the site. One of the vascularization strategies in tissue engineering applications is delivering vessel formation stimulators to the site of injury. Here, the focus is on the delivery of growth factors, cells, drugs, cell secretory substances, and other angiogenic and vascularization agents on the vessel and bone formation in CSDs.

Delivering GFs (such as VEGF, TGF-β, BMPs, and FGF), cells (EPCs and AdMSCs), drugs (such as puerarin), and cell secretory substances (such as exosomes) can improve the vascularization and angiogenesis in CSDs.

Moreover, to enhance vessel formation in CSDs, dual or combined delivery of angiogenic agents could be more promising because these agents can improve vessel formation in synergy. However, to stimulate both vessel and bone formation, a combination of angiogenic and osteogenic agents seems to be more promising.

Although better bone regeneration has been expected to be observed as a consequence of high vascularization in CSDs, the newly formed vascular system after treatment does not necessarily increase bone formation in a group of studies. Such contradistinction can be explained by the differences in GF type, release kinetics, delivery vehicle, implantation site, animal model, dose, study duration, and the type of vessels (type L or H) that have been formed.

## 5. Future Direction

Substantial advancements have been made to improve vessel formation in critical-sized bone defects using agent delivery techniques. However, for the future development of these agents, many avenues are remained to be examined.

Functionalization of the implant surface to improve vascularization and angiogenesis has great potential for further innovation since this field has not yet been widely investigated. Biofunctionalization of the implant surfaces using the dual delivery of cells or GFs can be considered the most established technique in improving vessel formation and consequent better bone formation. However, if they are to be implemented into manufacturing, these functionalization techniques are required to be adjusted to maintain the viability and activity of the agent.

When the biofunctionalization is designed using cells, this viability maintenance can be provided by in situ-based approaches, which are conducted directly in the operating room [117]. However, the effect of in situ cell delivery on the vascularization of CSDs has been rarely studied and seems to be a promising approach for future studies.

When it comes to the application of GFs, the sustained and localized delivery of GFs with short half-lives is still a barrier in tissue engineering applications [14]. To combat this limitation, localized gene delivery for prolonged times can be successfully used for the treatment of CSDs. Although gene delivery systems have been used in bone tissue engineering [118], limited studies have been conducted to study their effect on vessel formation in CSDs. In situ gene delivery systems [118] should be very useful for future studies to enhance vessel and bone formation in CSDs.

Further opportunities for innovation are presented in the use of large animal models. It is worth mentioning that different studies have been performed to assess the effect of different agents on bone formation in large animals’ CSDs [119]. However, large animal models have rarely been used to study the effect of agent delivery on vessel formation in CSDs. Using large animal models could possibly be very useful for future studies.

Targeting the newly discovered type H vessels is one of the other most promising future directions to improve bone formation in CSDs via enhancing vascularization.

## Figures and Tables

**Table 1:** The size of CSD according to the location in the body in different animal models.

Animal Model	Location	Size	Reference
Rat	Calvaria	5 mm diameter	[7,8]
Rat	Mandible	5 mm diameter	[9]
Rabbit	Femur	5 mm diameter, 6 mm height	[10]
Rabbit	Mandible	15 mm × 10 mm	[11,12]
Rabbit	Mandible	12 mm × 6 mm	[12]
Dog	Mandible	17 mm	[1]
Dog	Mandible	25 mm × 10 mm × 8 mm	[13]
Beagle	Tibia	2.0 cm and 1.6 cm segmental gaps	[14]
Sheep	Calvaria	20 × 20 × 5-mm	[15]
Sheep	Metatarsal	25 mm long	[16]

## Data Availability

There are no additional data available for this study other than what is reported in the manuscript.
